# Reassessment of the distinctive geometry of *Staphylococcus aureus* cell division

**DOI:** 10.1038/s41467-020-17940-9

**Published:** 2020-08-14

**Authors:** Bruno M. Saraiva, Moritz Sorg, Ana R. Pereira, Mário J. Ferreira, Léo C. Caulat, Nathalie T. Reichmann, Mariana G. Pinho

**Affiliations:** 1 Bacterial Cell Biology, Instituto de Tecnologia Química e Biológica António Xavier, Universidade Nova de Lisboa, Oeiras, Portugal; 2 Department of Biology, Ecole Normale Supérieure Paris-Saclay, Université Paris-Saclay, Gif-sur-Yvette, France; 3 Present Address: Department of Biochemistry, University of Oxford, Oxford, UK

**Keywords:** Cell division, Cellular microbiology

## Abstract

*Staphylococcus aureus* is generally thought to divide in three alternating orthogonal planes over three consecutive division cycles. Although this mode of division was proposed over four decades ago, the molecular mechanism that ensures this geometry of division has remained elusive. Here we show, for three different strains, that *S. aureus* cells do not regularly divide in three alternating perpendicular planes as previously thought. Imaging of the divisome shows that a plane of division is always perpendicular to the previous one, avoiding bisection of the nucleoid, which segregates along an axis parallel to the closing septum. However, one out of the multiple planes perpendicular to the septum which divide the cell in two identical halves can be used in daughter cells, irrespective of its orientation in relation to the penultimate division plane. Therefore, division in three orthogonal planes is not the rule in *S. aureus*.

## Abstract (web summary)

*Staphylococcus aureus* is thought to divide in three alternating orthogonal planes over three consecutive divisions. Here the authors dispel this idea, showing that one out of the multiple planes perpendicular to the septum can be used in daughter cells irrespective of its orientation in relation to the penultimate division plane.

## Introduction

Multi-drug resistant *Staphylococcus aureus* is a major cause of hospital acquired infections, as well as infections in the community setting that are becoming increasingly difficult to treat^1^. Besides its clinical relevance, *S. aureus* is also a good model to study cell growth and division of spherical cocci. Bacterial species with the suffix ‘cocci’ comprise species with near spherical cells, such as *S. aureus*, as well as the so-called ovococci such as *Streptococcus pneumoniae* or *Enterococcus faecalis*, which have an ovoid shape caused by peptidoglycan synthesis at the lateral wall, near the division site, that results in cell elongation^2^. We have recently shown that *S. aureus* cells are not perfectly spherical, as they undergo slight elongation mediated by the action of the penicillin binding protein PBP3, a peptidoglycan transpeptidase, and the SEDS (Shape, Elongation, Division and Sporulation) protein RodA proposed to be a peptidoglycan synthase with glycosyltransferase activity^3–6^.

While both rod-shaped bacteria and ovococci divide in successive parallel planes, perpendicular to the long axis of the cell, a distinctive characteristic of *S. aureus* division is that it is thought to occur in three alternating orthogonal planes over three consecutive division cycles. This mode of division was proposed in the 1970s on the basis of light microscopy images of individual *S. aureus* cells embedded in soft agar undergoing three consecutive divisions^7^ or scanning electron microscopy images of cubic packages of *S. aureus* cells grown in conditions that impair cell separation^8^. Division in three orthogonal planes requires that cells retain some form of ‘memory’ of the two previous division planes. However, the mechanism involved, possibly shared by other cocci with a similar mode of division such as *Micrococcus luteus* (shown to form cubic packets of cells when mutants impaired in cell separation were observed by scanning electron microscopy^9,10^), has remained elusive. Two models have been proposed for *S. aureus* division in three orthogonal planes, both based on the presence of perpendicular ‘scars’ of the previous divisions, present at the cell surface^11,12^. Turner and colleagues have shown that a large belt of peptidoglycan is formed at the division site which, after cell division, remains as orthogonal ribs that encode the location of previous divisions^12^ (Supplementary Fig. 1a). These structures could be used as epigenetic information to determine the orthogonality of the division planes over generations^12^. We have proposed that the junction between orthogonal ribs could be used as a geometric cue for the orientation of the axis of chromosome segregation^13^ (Supplementary Fig. 1b). *S. aureus* encodes the nucleoid occlusion protein Noc which preferentially binds to the origin proximal half of the chromosome and inhibits assembly of FtsZ, the first protein known to localize at the future division site^13–15^. As a consequence, progression of chromosome segregation releases midcell from Noc inhibition, allowing the FtsZ ring to be assembled at that position and therefore defining the plane of division^14,15^. Importantly, both models assume that scars of the two previous divisions divide the cell in quadrants. However, we have recently shown that upon cell division, the septum of a staphylococcal cell does not generate one hemisphere of each daughter cell, but only approximately one-third^4^. Therefore, the scar of a previous division is not placed at midcell, but off-centre^4^ (Supplementary Fig. 1c). This asymmetry makes it less likely that the peptidoglycan rib structures can be used as geometric cues to determine orthogonal division planes. We therefore questioned if *S. aureus* does indeed divide according to this geometry.

Here we use super-resolution fluorescence microscopy to show that although a plane of division is always perpendicular to the previous one, it is not necessarily perpendicular to the penultimate division plane. As a consequence, the majority of *S. aureus* cells do not divide in three alternating orthogonal planes.

## Results

### *S. aureus* does not necessarily divide in three orthogonal planes

**Fig. 1:**
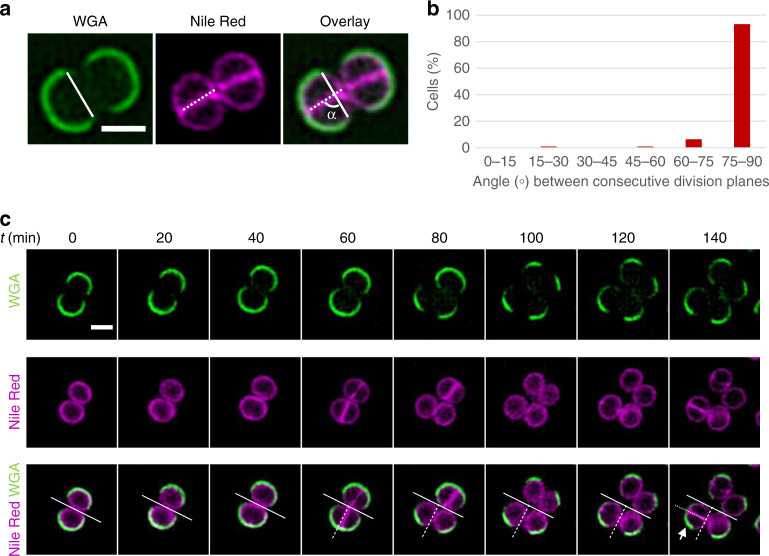
*S. aureus* cells divide in two, but not necessarily in three, alternating orthogonal planes. *S. aureus* strain COL cells were initially stained with cell wall dye WGA-488 (green) and membrane dye Nile Red (magenta). Excess of non-bound WGA-488 was removed by washing and Nile Red was added again before cells were placed on a medium-containing agarose pad and imaged every 20 min by structured illumination microscopy. **a** The border of the WGA-488-labelled region provides information on the orientation of the previous division plane (solid line), while the Nile Red-labelled septum indicates the orientation of the current division plane (dotted line). The angle (α) between the solid and dotted lines corresponds to the angle between the two consecutive planes of division. **b** Histogram of the angle α formed between two consecutive planes of division shows that these planes are orthogonal (with an average deviation from 90° of 6.7°, *n* = 183 cells examined over three biological replicates). Source data are provided as a Source data file. **c** First, second and third division planes are indicated by solid, dashed and dotted lines, respectively, showing that in the cell indicated by the arrow the third division plane is parallel, not perpendicular, to the first plane of division. Scale bars, 1 μm. A full field of view can be accessed in https://figshare.com/s/4808495d92a138aae36c.

To follow the planes of division of cells of the methicillin-resistant *S. aureus* (MRSA) strain COL, we labelled the membrane with Nile Red and the cell wall with a fluorescent derivative of wheat germ agglutinin (WGA-488), a lectin that binds N-acetylglucosamine residues present in the peptidoglycan and in teichoic acids of the *S. aureus* cell wall. After labelling, the excess of non-bound WGA-488 was removed by washing the cells with fresh medium, but Nile Red was maintained in the medium. Cells were placed on an agarose pad containing growth medium and imaged at 20 min intervals (Fig. 1 and Supplementary Fig. 2). While Nile Red diffuses over the cell membrane, the WGA-488 labelling is retained at its initial position on the cell wall and therefore can be used as a marker to follow the orientation of division planes. This is important because splitting of the septum of *S. aureus* cells occurs via an extremely fast (<2 ms) ‘popping’ event, often leading to two daughter cells connected by a hinge^4,16^ (Supplementary Fig. 2a). This movement, and the consequent change in relative position of the daughter cells, decreases the accuracy in determining the orientation of division planes if no marker besides the membrane dye is used.

Time-lapse images of dividing COL cells showed that each division plane is perpendicular to the previous one (Fig. 1a, b). However, we could observe various examples of cells that did not divide in three alternating orthogonal planes over three division cycles (see arrows in Fig. 1c and Supplementary Fig. 2b), suggesting that this mode of division is not always respected. Using this approach, we could quantify the number of cells dividing in two consecutive perpendicular planes (Fig. 1b), but we could not reliably determine the fraction of cells that did not divide in three consecutive alternating perpendicular planes, as daughter cells that result from divisions in a plane parallel to the microscopy slide become superimposed, often hindering imaging.

**Fig. 2:**
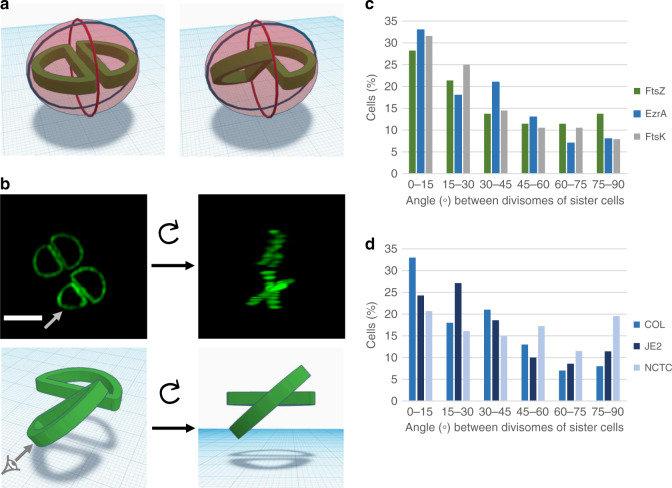
Sister cells can divide in different planes. **a** Model of cells undergoing division showing the previous (blue line), current (red line) and next (green D shape) division planes. Left image: if cells divide in three consecutive orthogonal planes then sister cells will inevitably divide in the same plane, perpendicular to the previous and current division planes. Right image: if the future division plane is not the same in both sister cells, then the orthogonal geometry of division is not respected in at least one of the sister cells, as exemplified by the left hemisphere where the next division will occur in a plane that is perpendicular to the current plane of division (in red) but not to the previous one (in blue). **b** Top panel: top view of 3D rendering of a Z-stack of images of COL EzrA-sGFP cells. Left panel shows the top view and right panel shows the view after rotating the 3D reconstruction to a perspective where division planes become perpendicular to the viewing plane, appearing as a cross. Scale bar, 1 µm. Bottom panel: model of the divisome of two sister cells, illustrating their orientation before and after rotation. Grey arrow indicates point of view after rotation. A full field of view can be accessed in https://figshare.com/s/907e54dd2d24e7c5dd05. **c**, **d** Histograms of angles formed by divisomes of sister cells showing that two sister cells very often select different planes of division. Measurements were performed using Z-stacks of images obtained from **c** COL strains expressing FtsZ^55-56^GFP (*n* = 131), EzrA-GFP (*n* = 100) or GFP-FtsK (*n* = 76) and **d** COL (*n* = 100), JE2 (*n* = 70) and NCTC (*n* = 87) strains expressing EzrA-GFP and analysed using automated software (see Methods). Two to four biological replicates were imaged for each strain. Quantifications were done from a single experiment for each strain. Source data are provided as a Source data file.

As an alternative approach to follow the orientation of division planes in *S. aureus*, we reasoned that if cells always divided in three alternating orthogonal planes, two sister cells would, inevitably, divide along the same plane, given that they share the same ‘history’ or ‘scar’ orientation from previous divisions (Fig. 2a, left panel). It follows that if two sister cells do not divide along the same division plane, then the distinctive geometry of division in three alternating orthogonal planes is not being adhered to in at least one of them (Fig. 2a, right panel). We observed that in *S. aureus*, the early divisome is assembled in a D shape in each of the two sister cells prior to septum splitting (Fig. 2b). Sister cells at this specific stage (still attached) allow a window of opportunity to image the exact orientation of the future division planes, before any eventual change in orientation occurs during cell splitting. The angle between two D-shaped divisomes in sister cells is therefore informative about the orthogonality of sequential division planes.

We labelled the early divisome of *S. aureus* using available fluorescent derivatives of three early cell division proteins, the tubulin homologue FtsZ^17^, the FtsZ regulator EzrA, a protein which directly interacts with FtsZ and is therefore often used as a proxy for its localization^17^, and the DNA translocase FtsK^18^. While the FtsZ fusion to GFP is not functional^17^ and was therefore expressed from a cadmium inducible promoter in a background that contains the native FtsZ protein, EzrA-GFP^3^ and GFP-FtsK^18^ fusions are functional, and were expressed from the native locus, under the control of the native promoter, in the absence of the corresponding native gene. 3D reconstructions of Z-stack images from dividing COL cells expressing one of these three fluorescent fusions clearly showed that the orientation of the divisome is often not the same in two attached sister cells (Fig. 2b). To manually measure the angle between the two division planes of sister cells, we rotated each 3D reconstruction to an orientation where division planes become perpendicular to the viewing plane, appearing as a cross (Fig. 2b, Supplementary Fig. 3 and Supplementary Movie 1). The smallest angle formed by the two divisomes was then measured for each pair of sister cells, where those dividing in the same plane would have an angle close to 0° (Supplementary Fig. 3a). These measurements indicated that for the majority of sister cells (>85%, *n* = 77), the two divisomes are not aligned (defined as divisomes at an angle larger than 15°). However, while for some cells angle measurement was straightforward (see examples in Supplementary Fig. 3b), for others, particularly when the angle was small, it was prone to user bias, as the orientation of the two divisomes was difficult to assess (see examples in Supplementary Fig. 3c). We therefore developed a script to automatically measure the angles between divisomes by generating kymographs to convert the 3D information into 2D data (see Methods and Supplementary Figs. 4 and 5). This method enabled us to eliminate user bias, and so was used for subsequent analysis.

As seen in Fig. 2c, divisomes in sister cells of *S. aureus* strain COL can be placed at any angle from 0 to 90°. Although there was some preference for smaller angles, the majority of cells with a complete septum had the divisomes of the two future daughter cells placed at angles larger than 15° (>~70%, *n* = 307). This observation was independent of the fluorescent fusion used to label the divisome (FtsZ^55-56^GFP, EzrA-GFP or GFP-FtsK) and therefore it is unlikely to be an artefact due to the presence of the fluorescent proteins. It is also not due to free movement of the divisome, as we showed that once this structure is assembled, its orientation is essentially maintained (Supplementary Fig. 6).

To determine if the mode of division observed for COL was common to other *S. aureus* strains, we determined the placement of the divisome in the community-acquired MRSA strain JE2 and in methicillin susceptible *S. aureus* (MSSA) strain NCTC8325-4, using EzrA-GFP to localize the divisome (Fig. 2d). Like in strain COL, the majority of sister cells in NCTC8325-4 or JE2 strains (>75%, *n* = 87 and *n* = 70, respectively) had the divisomes placed at an angle larger than 15°, clearly indicating that division in three alternating orthogonal planes over consecutive generations is often not respected in all tested *S. aureus* strains.

## Discussion

Cell division in three consecutive orthogonal planes has been considered a hallmark of *S. aureus*, ever since it was proposed, over four decades ago^7,8^. This model was based on scanning electron microscopy images, which do not allow analysis over consecutive division cycles, or on phase contrast microscopy images of dividing *S. aureus* cells, where the geometry of division can only be followed based on the relative position of daughter cells, which is difficult to determine with precision, mainly when cells divide in the plane of the microscope slide. This geometry of division in alternating orthogonal planes implies ‘memory’ of the two previous division planes, and therefore it suggests that *S. aureus* cells should have a specific molecular mechanism to ensure this mode of division, which we and others have tried, without success, to identify. Using super-resolution structured illumination microscopy and acquiring Z-stacks that provide 3D information of the division planes, we have now shown that such a mechanism is not required by staphylococcal cells, as they often do not divide in three consecutive alternating orthogonal planes (see video at https://youtu.be/2Bw-SKu7pbQ for an animation of three consecutive division cycles). It is important to note, however, that each division plane is always perpendicular to the previous plane, although not necessarily to the one before that. Cell division in two perpendicular planes has been proposed for other cocci. In *Neisseria gonorrhoeae* this mode of division is ensured by the Min system, a nucleoid-independent system to position the cell division plane through localized inhibition of FtsZ assembly^19^. Although the localization of gonococcal Min proteins is unknown, expression of GFP-tagged *N. gonorrhoeae* MinD in round (*rodA*-deficient) *E. coli* cells has shown that the protein oscillates in a plane parallel to the complete septum^20^. This movement should generate a region with minimal MinD concentration in a plane perpendicular to the previous septum, making it the most probable division site^20^. A similar mechanism has been recently proposed for division of *Deinococcus radiodurans* in two perpendicular planes^21^.

**Fig. 3:**
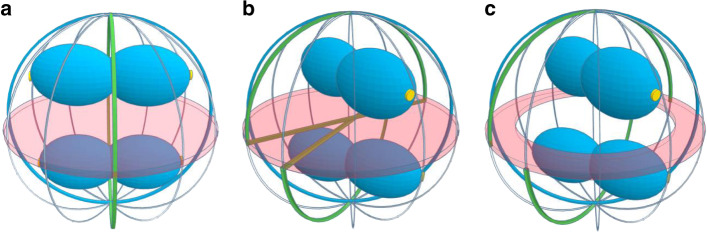
New model for the geometry of cell division in *S. aureus*. Scheme of dividing *S. aureus* cells showing the scar of the previous division plane (blue ring), the current division plane (red disk) and the localization of the future division plane, where the divisome assembles (green ring). Chromosomes are shown in blue with origins of replication shown as small yellow discs. **a** The axis of chromosome segregation, which is parallel to the closing septum, establishes the division plane in *S. aureus*, as the origin proximal region is bound by the nucleoid occlusion effector Noc, which prevents FtsZ assembly. The only division plane that does not lead to bisection of the nucleoid is the plane indicated in green. **b** Chromosome segregation towards any of the meridians (grey lines) is geometrically equivalent. Therefore, chromosomes in sister cells can segregate in different orientations, releasing different planes for division (green lines). **c** Chromosome segregation can precede septum formation suggesting that positioning of the chromosomes, and not only of the septum, may prevent chromosome segregation towards the poles.

However, *S. aureus* does not have a Min system, and division in two perpendicular planes is instead dictated by the nucleoid occlusion effector Noc^13^, which preferentially binds the origin proximal region of the chromosome and prevents FtsZ polymerization over that region of the chromosome^15^. Therefore, once the axis of chromosome segregation is established, there is only one possible plane of division that does not bisect the nucleoid (green circumference in Fig. 3a). The role of chromosome segregation in driving division site selection has been also proposed in the ovococci *S. pneumoniae*, which interestingly lacks not only a Min system but also a nucleoid occlusion effector^22^. Chromosome segregation in *S. aureus* likely occurs along an axis parallel to the closing septum, as segregation perpendicularly to the septum (towards the ‘poles’) would be less favourable for chromosome demixing due to spatial constraints^23^. It follows that a plane of division that does not bisect the nucleoid is necessarily perpendicular to the previous division plane (red disk in Fig. 3a) and so no other mechanism is in principle required to ensure division in two perpendicular planes. However, segregation towards any of the ‘meridians’ (grey lines in Fig. 3) is geometrically equivalent, as long as the cell is circular at the division plane. We now propose that the choice of axis of segregation is essentially random, as long as it is parallel to the closing septum. As a consequence, (i) each division plane is always perpendicular to the previous one, but not necessarily to that of two divisions ago (blue circumferences in Fig. 3) and (ii) sister cells can, and often do, choose different division planes. Nevertheless, it is possible that some cells have a small geometric deformation, leading to a septum that is not a perfect circle, which could favour a specific orientation of the chromosomes in both sister cells, along a slightly longer axis, explaining the preference seen towards small angles particularly in strains COL and JE2.

Interestingly, we observed that, in a fast-growing strain, chromosome segregation can start before completion of the previous closing septum (scheme in Fig. 3c and Supplementary Fig. 7). We observed cells in which an early divisome was present (assessed by the presence of an open EzrA or FtsZ ring) but with no signs of membrane invagination at the division plane, and two separated chromosome replication origins could already be seen in one of the future daughter cells (see cells marked with an asterisk in Supplementary Fig. 7). Therefore, it is possible that it is not the division septum that constrains a chromosome to one daughter cell and impairs segregation towards the ‘poles’, but the physical presence of the other chromosome.

Finally, we show for the first time, to the best of our knowledge, that FtsZ can initially assemble as a ‘D’ shaped structure, instead of as a ring, in live bacterial cells. Non-ring shapes of FtsZ have been previously observed in sculpted *E. coli* cells forced into unnatural shapes, including squares, hearts, stars and also ‘D’ shape^24^. This implies that the curvature of the FtsZ polymers is not constant around the cell and that the shape of the FtsZ structure may be dictated by the bacterial cell shape. It will be interesting to determine in the future if the dynamics of FtsZ polymers is affected by the curvature of the Z-ring and what impact the rapid *S. aureus* popping has on the assembling divisome.

## Methods

### Strains and growth conditions

Strains used in this study are listed in Supplementary Table 1. *S. aureus* strains were grown in tryptic soy broth (TSB, Difco) at 200 rpm with aeration at 37 °C or on tryptic soy agar (TSA, VWR) at 37 °C. When necessary, culture media was supplemented with antibiotics (10 μg ml^−1^ erythromycin or 50 μg ml^−1^ kanamycin and 50 μg ml^−1^ neomycin), with 100 μg ml^−1^ 5-Bromo-4-chloro-3-indolyl β-D-galactopyranoside (X-gal, Apollo Scientific), with 0.1 mM isopropyl β-D-1-thiogalactopyranoside (IPTG, Apollo Scientific) or with 0.1 µM cadmium chloride (Sigma-Aldrich).

To introduce the *ezrA-sgfp* fusion in different *S. aureus* strains, pMAD-ezrAsgfp^3^ was transduced into strains COL, NCTC8325-4 and JE2, using phage 80α as previously described^25^. Integration–excision of the plasmid resulted in the introduction of a 3′ *sgfp* fusion to the native copy of the *ezrA* gene. To construct COL *sfgfp-ftsK*, the plasmid pBCBHV017^18^ was transduced into COL followed by integration–excision which resulted in the introduction of a 5′ *sgfp* fusion to the native copy of the *ftsK* gene.

To obtain strain BCBAJ096, the integrative plasmid pBCBHV004^13^ (encoding a *spo0J-yfp* fusion) was introduced into strain BCBAJ012^26^ (COL *ezrA:ezrA-mCherry*). The expression of *spo0J-YFP* in BCBAJ096 is under the control of the native promoter while a second copy of *spo0J* is under the control of the *Pspac* promoter.

### Microscopy

For time-lapse microscopy, overnight cultures of strain COL were back-diluted 1:500 in TSB and grown to mid-exponential phase (OD_600nm_ ≈ 0.5). One millilitre of the culture was incubated for 5 min with the membrane dye Nile Red (5 µg ml^−1^, Invitrogen) and the cell wall dye wheat germ agglutinin conjugated to Alexa Fluor488 (WGA-488 1 µg ml^−1^, Invitrogen), subsequently pelleted and resuspended in 20 µl TSB containing Nile Red (5 µg ml^−1^, Invitrogen). One microlitre of the labelled culture was then placed on a microscope slide covered with a thin layer of agarose (1.2% (w/v) in 1:1 phosphate buffered saline (PBS)/TSB solution). Time-lapse images were acquired every 20 min by structured illumination microscopy (SIM) in an Elyra PS.1 microscope (Zeiss) using a Plan-Apochromat 63x/1.4 oil DIC M27 objective. SIM images were acquired using three grid rotations, with 34 μm grating period for the 561 nm laser (100 mW) and 28 μm period for 488 nm laser (100 mW). Images were captured using a Pco.edge 5.5 camera and reconstructed using ZEN software (black edition, 2012, version 8.1.0.484) on the basis of a structured illumination algorithm^27^.

For visualization of divisome proteins, overnight cultures of *S. aureus* strains were back-diluted 1:500 in TSB (supplemented with CdCl_2_ in the case of ColFtsZ^55-56^sGFP) and allowed to grow to an OD_600nm_ of ~0.8. One millilitre of each culture was pelleted and resuspended in 20 µl PBS. One microlitre of this culture was then placed on a microscope slide covered with a thin layer of agarose (1.2% (w/v) in PBS). Z-stacks of 20–25 images, with a 110-nm increment, were acquired by SIM using an Elyra PS.1 microscope (Zeiss) and a 488-nm laser, as described above.

For time-lapse microscopy of fluorescent protein fusions with acquisition of a Z-stack at each time point (Supplementary Fig. 6), cultures of COL EzrA-sGFP or ColFtsZ^55-56^sGFP were grown to exponential phase in TSB or TSB supplemented with CdCl_2_, respectively. One millilitre of each culture was pelleted and resuspended in 30 µl of TSB. One microlitre of this culture was placed on a microscope slide covered with a thin layer of agarose (1.2% (w/v) in PBS:TSB in a proportion of 3:2), incubated at 37 °C in a DeltaVision OMX SR microscope (GE). Z-stacks of 19 images, with a Z step of 125 nm were acquired every 3 min by SIM using an Olympus 60X PlanApo N/1.42 oil objective. A 488-nm laser (100 mW) was used at 11–18 W cm^−2^ with an exposure time of 10 ms. The software AcquireSRsoftWoRx (GE) was used for image acquisition and reconstruction of the SIM images.

To image strain BCBAJ096, which expresses fluorescent derivatives of EzrA and Spo0J (fused to mCherry and YFP, respectively), cells were grown in TSB containing erythromycin and IPTG to an OD of 0.8. To label the DNA, 1 ml of the culture was incubated with the DNA dye Hoechst 33342 (1 µg ml^−1^) for 5 min, pelleted and resuspended in 30 µl of PBS. Cells (1 µl) were placed on a thin layer of agarose (1.2% (w/v) in PBS) mounted on a microscope slide. Z-stacks of 21 epifluorescence images with a Z step of 125 nm were acquired using a DeltaVision OMX SR microscope (GE) with an Olympus 60X PlanApo N/1.42 oil objective. The fluorophores were excited with a 488-nm laser (100 mW), a 568-nm laser (100 mW) and a 405-nm laser (100 mW). The software AcquireSRsoftWoRx (GE) was used for image acquisition and deconvolution.

To image strain BCBHV005, which expresses fluorescent derivatives of FtsZ and Spo0J (fused to CFP and YFP, respectively), cells were grown in TSB supplemented with IPTG, kanamycin, neomycin and erythromycin to mid-exponential phase. One millilitre of cell culture was labelled with the membrane dye FM5-95 (250 ng ml^−1^, Invitrogen) for 5 min, pelleted and resuspended in 20 µl PBS. Cells were placed on a thin layer of agarose (1.2% (w/v) in PBS) and imaged using a Zeiss LSM 880 inverted confocal laser scanning microscope with a Plan-Apochromat 63x/1.4 Oil DIC M27 objective. For YFP visualization, a 514 nm argon laser (25 mW) was used for excitation and wavelengths between 518 and 566 nm were detected using a gallium arsenide phosphide (GaAsP) detector. CFP and FM5-95 were imaged simultaneously; CFP was excited with a 458-nm argon laser (25 mW) and wavelengths between 462 and 556 nm were detected with a GaAsP detector, while FM5-95 was excited with a 561-nm laser (20 mW) and wavelengths between 650 and 758 nm were detected with a photomultiplier tube (PMT) detector. The gain was set to 700 for all detectors and the Zeiss Zen 2.3 (black edition) software was used for image acquisition. The smooth option of FIJI^28^ was applied to the images.

### Analysis of planes of division over two generations

The relative angle between consecutive planes of division was measured with FIJI^28^ using time-lapse images of COL cells labelled with both WGA-488 and Nile Red. WGA-488 was used to determine the orientation of the previous division plane, defined as the line that connects the two ends of WGA-488 labelled region in each cell (see Fig. 1a). Nile Red was used to determine the orientation of the current plane of division. The smallest angle between the plane of the septum and the previous division plane was measured for each cell. The average difference to 90° was also calculated.

### Analysis of the orientation of divisomes in sister cells

The divisomes of two attached sister cells in dividing *S. aureus* cells showing a closed septum were visualized using GFP fluorescent derivatives of the divisome proteins FtsZ, EzrA or FtsK. The angle between divisomes assembled in attached sister cells was measured using kymographs, generated using FIJI^28^, obtained from Z-stacks of fluorescence images, by drawing a line crossing or overlapping the divisome of each sister cell, parallel to the flat region adjacent to the septum (see Supplementary Fig. 4a, b). A binary image of the kymograph was generated using a threshold corresponding to the 95 percentile of all pixel intensities. The coordinates of the pixels with an intensity above the threshold (considered as signal) were extracted and used as input data for a principal component analysis (PCA)^29^. The first principal component, corresponding to the eigenvector with highest eigenvalue, was used as the vector that defines the orientation of fluorescence signal in the kymograph. Two vectors were generated for each pair of sister cells (*V*_1_ and *V*_2_, for sister cells 1 and 2, respectively, see Supplementary Fig. 4c). The slope of the vector was corrected to account for the pixel size of the camera and the acquisition step between each Z-plane, by multiplying the *x* coordinates by the pixel size and the *y* coordinates by the Z step. Vectors with a positive slope were considered to generate positive angles while vectors with negative slope generated negative angles. The smallest angle of *V*_1_ and *V*_2_ relative to the axis corresponding to the plane of microscopy imaging was measured for each pair of sister cells and named *α*_1_ and *α*_2_ (Supplementary Fig. 4c). The amplitude of the angle between the divisomes of pairs of sister cells was calculated as the absolute value of the difference between *α*_1_ and *α*_2_.

### Reporting summary

Further information on research design is available in the Nature Research Reporting Summary linked to this article.

## Supplementary information

Supplementary Information

Description of Additional Supplementary Files

Supplementary Movie 1

Supplementary Software

Reporting Summary

## Data Availability

The datasets generated during the current study are available as a source data file and from the corresponding author on request. Original images for Figs. 1 and 2 are available at figshare (https://figshare.com/s/4808495d92a138aae36c and https://figshare.com/s/907e54dd2d24e7c5dd05, respectively). Source data are provided with this paper.

## Source data

Source data

## Code availability

Code is available as Supplementary Software, and also from github (https://github.com/BacterialCellBiologyLab/KymographsAngleMeasurement).
