# Supplementary material for: Reassessment of the distinctive geometry of Staphylococcus aureus cell division
Source: Nat Commun. 2020 Aug 14;11:4097. doi: 10.1038/s41467-020-17940-9 (PMC7427965; doi:10.1038/s41467-020-17940-9)
Supplement: Supplementary file 1 — Supplementary Information [file 41467_2020_17940_MOESM1_ESM.pdf]

Supplementary Information

Reassessment of the distinctive geometry of *Staphylococcus aureus* cell division

Saraiva, Sorg *et al*

Supplementary Table 1. Strains used and constructed in this study

Strains	Description	Source or reference
RN4220	Restriction-negative derivative of NCTC8325-4	1
NCTC8325-4	MSSA strain	R. Novick
USA300 JE2	CA-MRSA	2
COL	HA-MRSA	3
8325-4*sfgfp-ftsK*	NCTC8325-4 *ftsK::sfgfp-ftsK*	4
COL *sfgfp-ftsK*	COL *ftsK::sfgfp-ftsK*	This work
COL EzrA-sGFP	COL *ezrA::ezrA-sgfp*	This work
ColFtsZ$^{55\text{-}56}$sGFP	COL pCN-ftsZ$^{55\text{-}56}$sGFP; Kan^r	5
JE2 EzrA-sGFP	JE2 *ezrA::ezrA-sgfp*	This work
NCTC EzrA-sGFP	NCTC *ezrA::ezrA-sgfp*	This work
BCBHV005	RN4220 *ftsZ*::pBCBHV006 *spo0J*:: pBCBHV004, expressing *ftsZ-cfp* and *spo0J-yfp* fusions; Kan^r, Ery^r	6
BCBAJ012	COL *ezrA::ezrA-mCherry*	7
BCBAJ096	COL *ezrA::ezrA-mCherry spo0J*:: pBCBHV004, expressing *spo0J-yfp*; Ery^r	This work

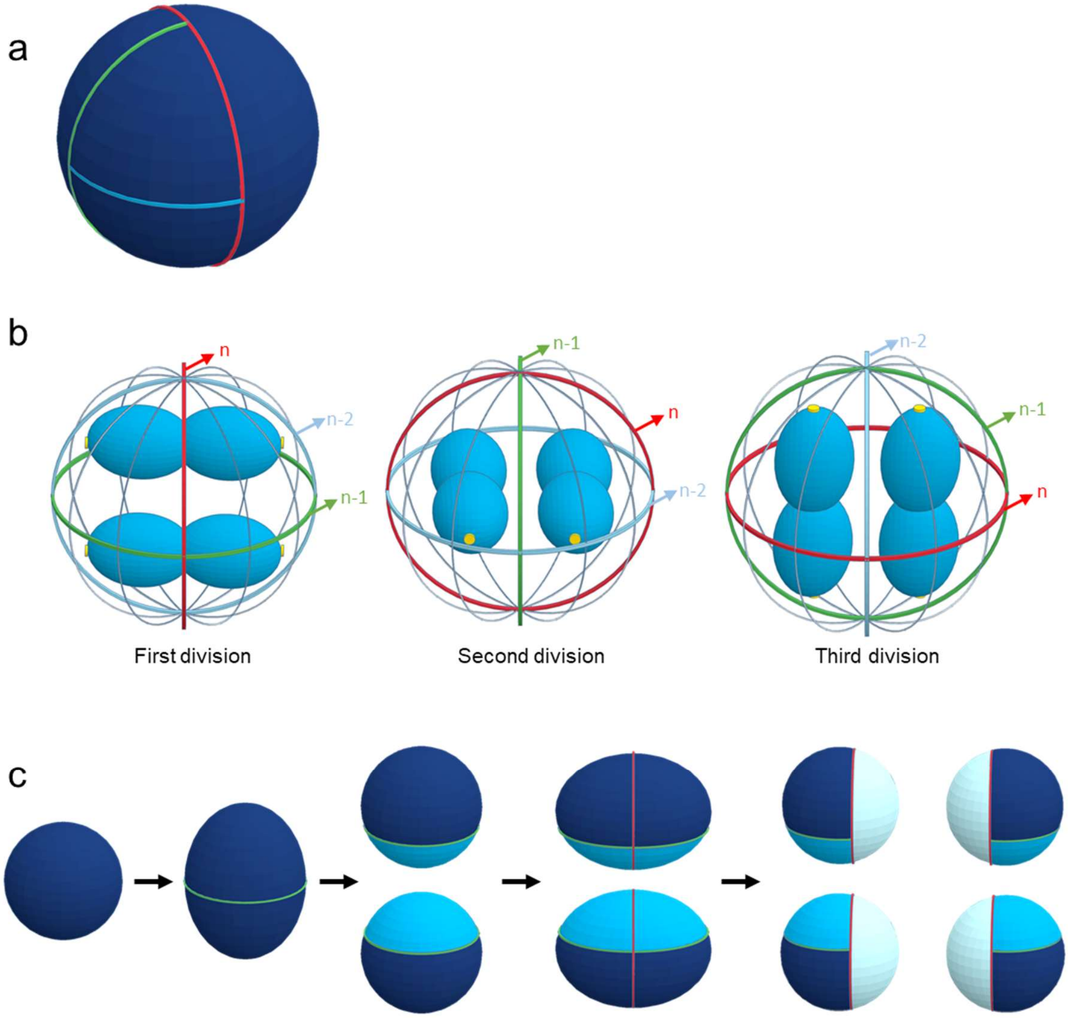

Supplementary Figure 1. Specific peptidoglycan structures at the cell surface could provide epigenetic information to determine future division sites in *S. aureus*. (a) Schematic representation of the cell wall surface of *S. aureus* showing scars of previous division planes that result from the formation of large belts of peptidoglycan at the division site, before septum formation. The right hemisphere corresponds to new peptidoglycan, originating from the septum of the mother cell, and therefore contains no scars. The left hemisphere contains scars from the last three rounds of division indicated by red (most recent), green and blue lines, all perpendicular to each other. These scars could encode the required epigenetic information to divide accurately in three alternating perpendicular planes (based on Ref.[8]). **(b)** Illustration of *S. aureus* cell showing the orientation of the current division plane (n, red), the previous one (n-1, green) and that from two divisions ago (n-2, blue). Chromosomes are shown in blue with origins of replication shown as small yellow disks. This model assumes that chromosomes segregate towards the junction of the two previous division planes (cross between blue and green circles). This releases only one possible division plane (red) that does not bisect the nucleoid. With each division, each scar ages by one generation, resulting in division in three alternating orthogonal planes (based on Ref.[6]). **(c)** Depiction of two consecutive divisions in orthogonal planes. Newly divided cells are approximately spherical but become slightly elongated during cell cycle progression. After the first cell division, the

surface cell wall composition of each daughter cell consists of approximately two thirds of old peptidoglycan (dark blue) and approximately one third of newly synthesized peptidoglycan. As the septum does not originate one complete hemisphere of each daughter cell, the peptidoglycan scars are not placed in the middle of the cell, but are off center (based on Ref.[9]).

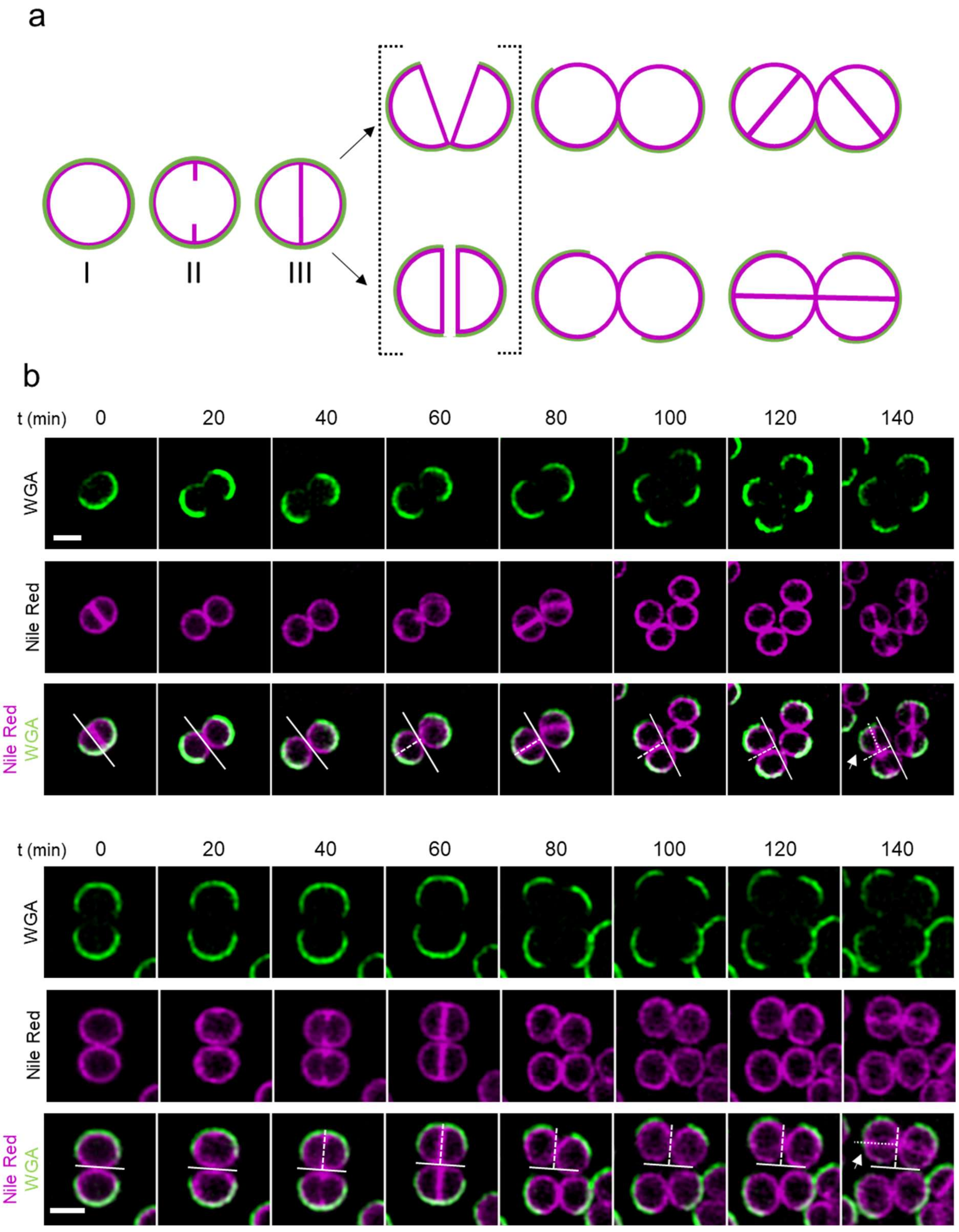

Supplementary Figure 2. Examples of *S. aureus* cells that did not divide in three alternating orthogonal planes over three division cycles. **(a)** Schematic representation of *S. aureus* cell cycle depicting cells initially stained with cell wall dye WGA-488 (green) and membrane dye Nile Red (magenta) where excess of non-bound WGA-488 is removed by washing and Nile Red is added again before cells are placed on medium-containing agarose pad and allowed to

grow. The stage of the cell cycle can be assessed based on the membrane dye labelling, showing cells with no septum (I), a forming septum (II) or a complete septum (III), which is rapidly split in a process that takes only a few milliseconds (cells in dashed brackets). This process can lead to changes in the orientation of the daughter cells, leading to two daughter cells connected by a hinge (top). In other cases, cells retain their relative orientation after splitting (bottom). **(b)** *S. aureus* strain COL cells were labelled as described above and imaged every 20 min by structured illumination microscopy. First, second and third division planes are indicated by solid, dashed and dotted lines, respectively, showing that in cells indicated by arrows the third division plane is parallel, not perpendicular, to the first plane of division. Scale bars, 1 µm.

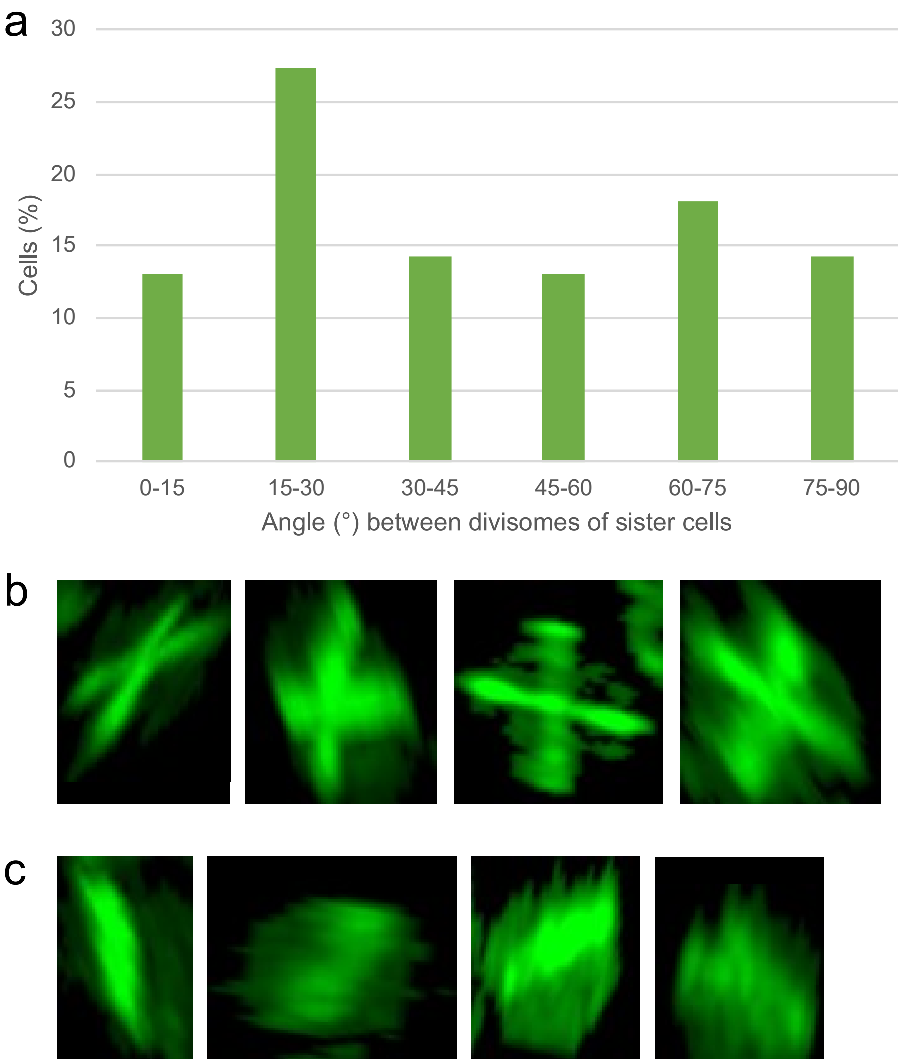

Supplementary Figure 3. Manual measurement of angles between sister cells division planes shows that *S. aureus* cells do not necessarily divide in three alternating orthogonal planes. **(a)** Histogram of angles formed by divisomes of sister cells showing that two sister cells very often select different planes of division. Manual measurements of angles were performed on rotated 3D renders of Z-stacks of ColFtsZ$^{55\text{-}56}$sGFP images (n=77, from one experiment). Source data are provided as a Source Data file. **(b)** Examples of 3D reconstructions of Z-stacks of images of strain ColFtsZ$^{55\text{-}56}$sGFP rotated so that division planes become perpendicular to the viewing plane, used to manually measure the angle of divisomes in sister cells. **(c)** Examples of rotated 3D reconstructions that were discarded for angle measurements, due to the low resolution after rotation of the 3D rendering, impairing accurate assessment of the relative position of each division plane.

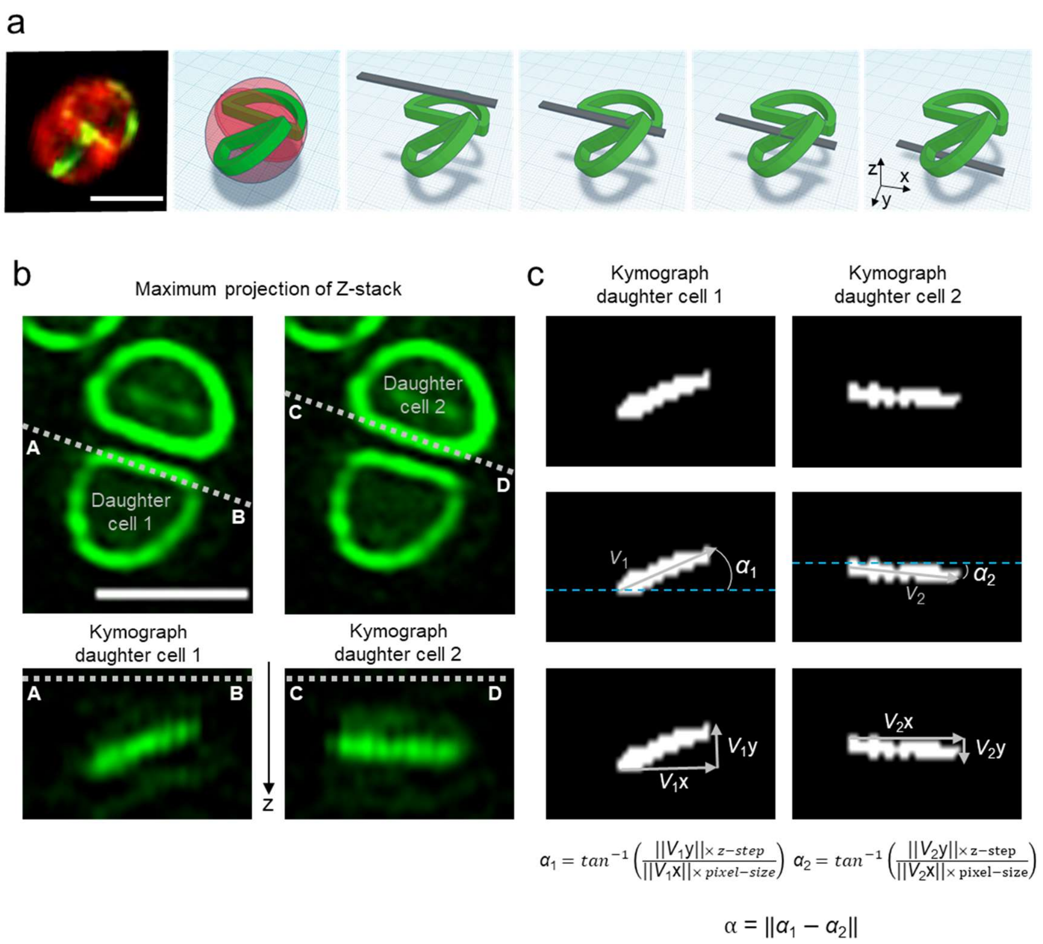

Supplementary Figure 4. Strategy to measure the angle between division planes of two sister cells using Z-stacks kymographs. **(a)** The left image shows a cell of strain COL *sfgfp-ftsK* labelled with membrane dye FM5-95 and imaged by SIM, where the septum has been formed and the divisome has assembled in each sister cell (scale bar 1 µm); the second image shows a model of that cell where the divisomes are seen as green D-shapes; the next four images illustrate the process used to generate the kymographs in panel b, where each line corresponds to the fluorescence signal obtained for a Z plane (grey rectangle) of a 3D reconstruction of the cells encoding a fluorescent derivative of a divisome protein (FtsZ, EzrA or FtsK). **(b)** Maximum projection of a Z-stack (top) obtained by imaging a single dividing cell (containing two attached sister cells) of strain ColFtsZ$^{55\text{-}56}$sGFP and corresponding kymographs of the divisome of each sister cell (bottom). Kymographs were generated by tracing two lines (dotted) that cross/overlap the divisome of each sister cell and are parallel to its flat region. Scale bar, 1 µm. **(c)** To measure the angle formed between the divisome of each sister cell, the fluorescence signal in the kymographs was first converted to a binary image (top panel, see methods); a vector indicating the orientation of the kymograph line (V_1 and V_2, for sister cell 1 and 2, respectively) was then calculated by using the coordinates of the white pixels as input for a Principal Component Analysis (PCA) and choosing the first component as the orientation vector. The angle between this vector and the microscopy imaging plane (dashed blue line) is indicated as α_1 and α_2, for sister cell 1 and 2, respectively (middle panel). The bottom panel shows the x and y components of V_1 and V_2 used to calculate

α_1 and α_2. This was done by determining the arctangent of the ratio between the norms of Vx and Vy. Norms of Vx were corrected by multiplying by the camera pixel size. Norms of Vy were corrected by multiplying by the step size between each plane of the Z-stack. The angle α between the divisome of the two daughter cells is calculated as the absolute difference between α_1 and α_2, as indicated in the equation.

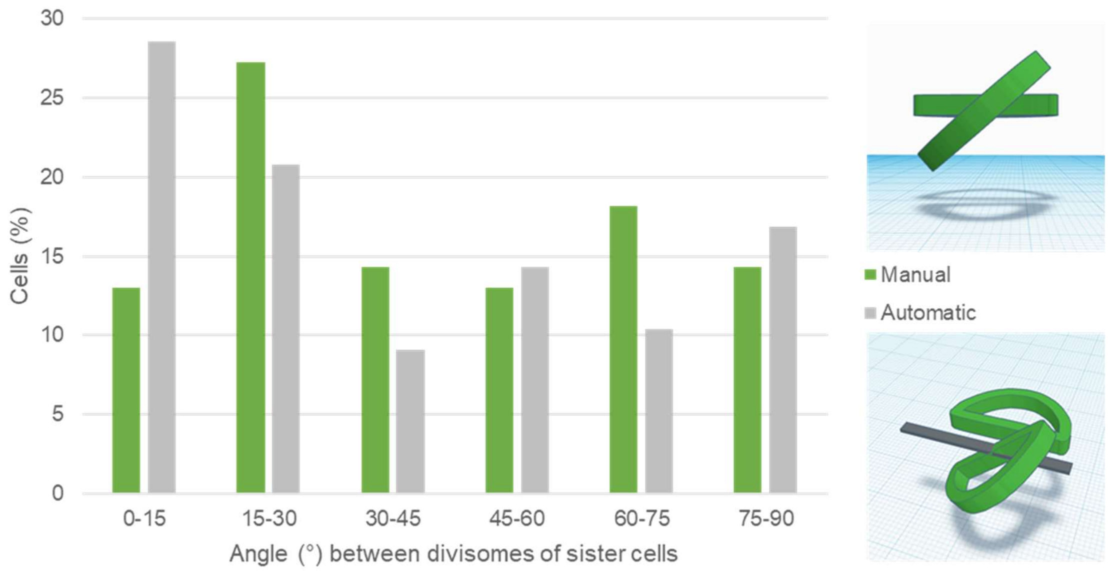

Supplementary Figure 5. Manual and automatic kymographs show similar angle distribution. Histograms of angles formed by divisomes of sister cells of strain ColFtsZ$^{55-56}$sGFP measured both manually (green) and with automated software (grey), n=77 from one experiment. Both methods show that sister cells can divide in different planes. Source data are provided as a Source Data file.

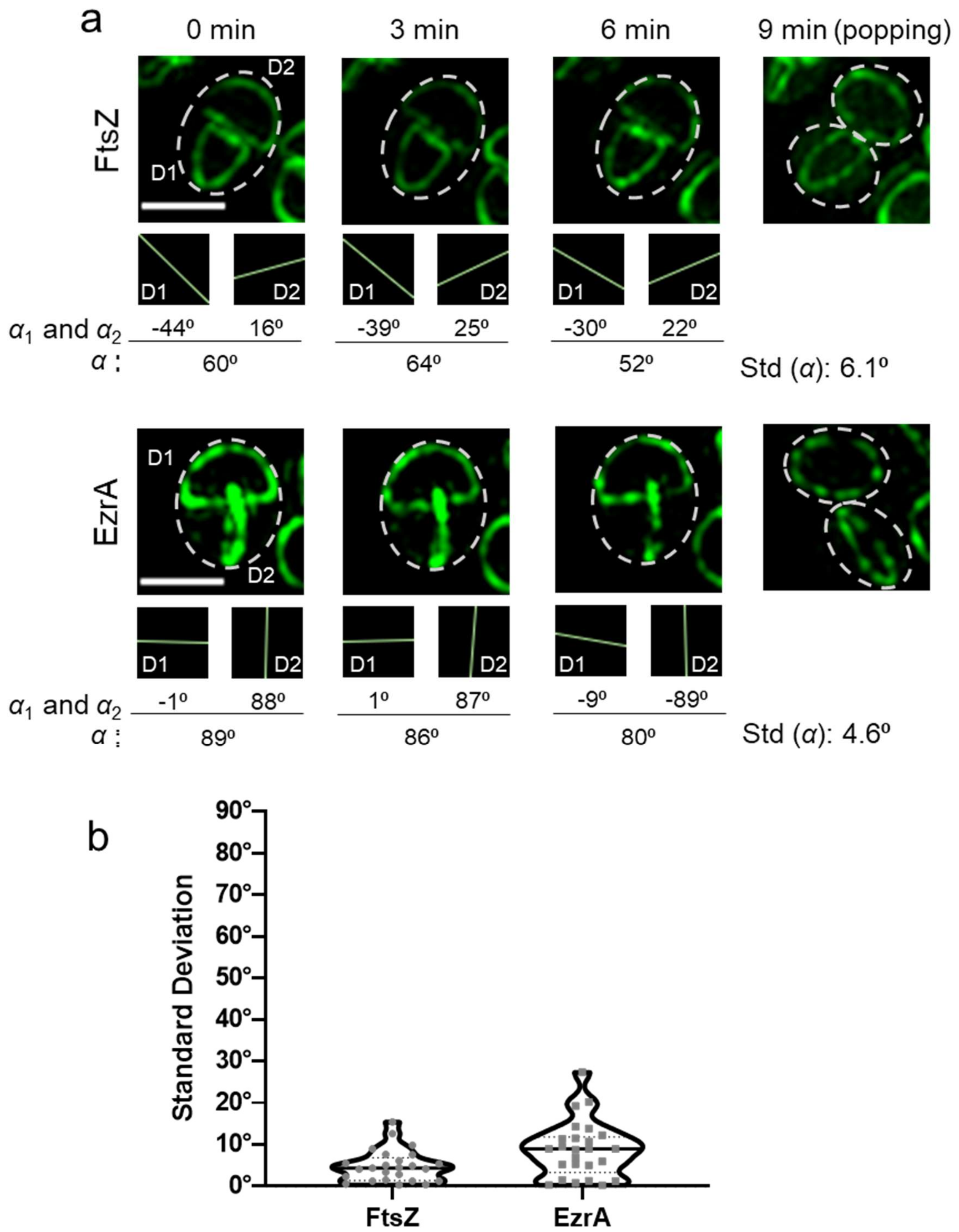

Supplementary Figure 6. Assembled *S. aureus* divisomes do not freely move/rotate. **(a)** Examples of timelapse images (maximum projections of Z-stacks) of strains ColFtsZ$^{55\text{-}56}$sGFP (top) and COL EzrA-sGFP(bottom) acquired every 3 minutes by SIM. Dashed lines indicate the outline of cells. In both examples shown, cells divided and "popped" between 6 and 9 min timepoints. Squares with green lines indicate orientation of the divisomes in two daughter cells (D1 and D2), determined as described in Supplementary Figure 4. α_1 and α_2 correspond to the angle of the divisome of daughter cells D1 and D2 to the microscopy imaging plane. α

corresponds to the angle between the divisomes of the two daughter cells D1 and D2. If a cell moves on the slide during the timelapse, the absolute values of α_1 and α_2 will vary, but α should remain constant, unless the two divisomes move relatively to each other. Scale bars, 1 µm. **(b)** Violin plots where each data point corresponds to the standard deviation of the angles α obtained at different time points for one cell, for strains ColFtsZ$^{55\text{-}56}$sGFP (left, n=25 cells examined from two biological replicates) and COL EzrA-sGFP (right, n=25 cells examined from two biological replicates). Solid line indicates median and dashed lines indicate 25 and 75 percentiles. Source data are provided as a Source Data file.

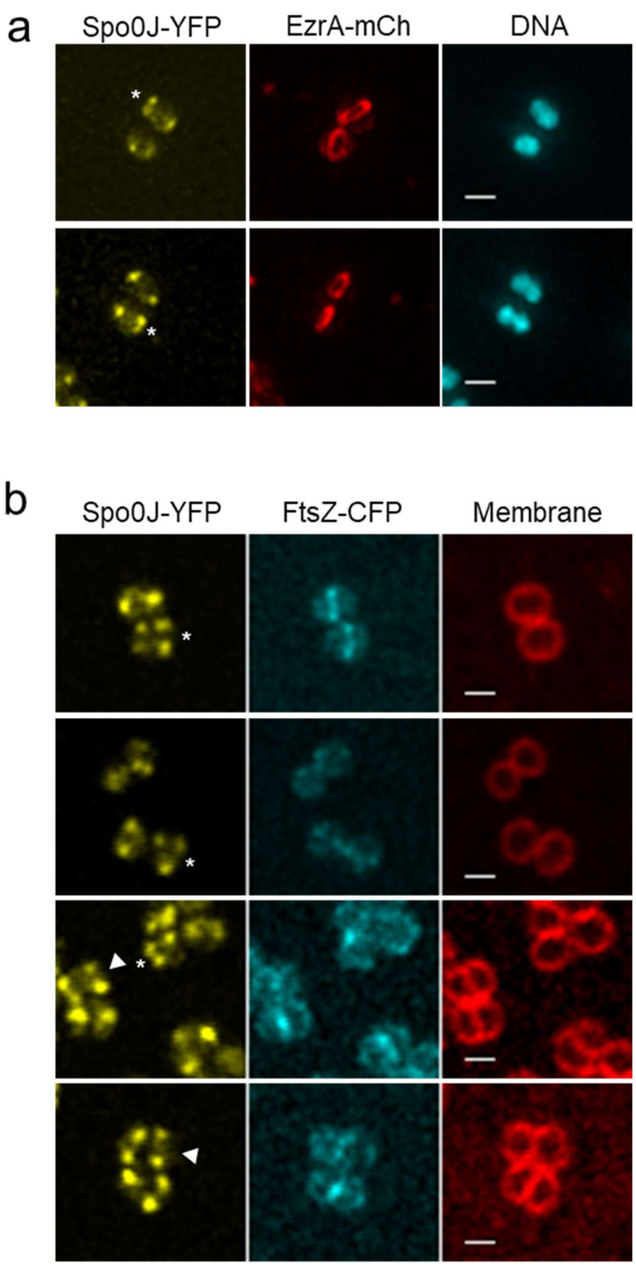

Supplementary Figure 7. Chromosomes segregation precedes divisome assembly in daughter cells. (a) Maximum projection of Z-stacks of deconvolved epifluorescence images of strain BCBAJ096 (COL expressing EzrA-mCherry and Spo0J-YFP fusions), labelled with the DNA dye Hoechst 33342. Spo0J binds near the chromosome origin of replication, so Spo0J-YFP localization can be used as a proxy for origin localization. Asterisks show cells where chromosome segregation in one of the daughter cells is observed prior to closure of the divisome ring. **(b)** Confocal images of strain BCBHV005 (RN4220 expressing FtsZ-CFP and Spo0J-YFP fusions) labeled with the membrane dye FM5-95. Arrowheads show cells where chromosome segregation precedes divisome assembly in attached sister cells. Asterisks show cells where two origins can be observed in at least one of the future sister cells before any sign of membrane invagination at the division plane (11% of pre-septal cells, n=430 cells examined from three biological replicates). Scale bars, 1 µm.

Supplementary References

1 Nair, D. *et al.* Whole-genome sequencing of *Staphylococcus aureus* strain RN4220, a key laboratory strain used in virulence research, identifies mutations that affect not only virulence factors but also the fitness of the strain. *J Bacteriol* **193**, 2332-2335, doi:10.1128/jb.00027-11 (2011).

2 Fey, P. D. *et al.* A genetic resource for rapid and comprehensive phenotype screening of nonessential *Staphylococcus aureus* genes. *mBio* **4**, e00537-00512, doi:10.1128/mBio.00537-12 (2013).

3 Gill, S. R. *et al.* Insights on evolution of virulence and resistance from the complete genome analysis of an early methicillin-resistant *Staphylococcus aureus* strain and a biofilm-producing methicillin-resistant *Staphylococcus epidermidis* strain. *J. Bacteriol.* **187**, 2426-2438, doi:10.1128/JB.187.7.2426-2438.2005 (2005).

4 Veiga, H. & M, G. P. *Staphylococcus aureus* requires at least one FtsK/SpoIIIE protein for correct chromosome segregation. *Mol Microbiol* **103**, 504-517, doi:10.1111/mmi.13572 (2017).

5 Monteiro, J. M. *et al.* Peptidoglycan synthesis drives an FtsZ-treadmilling-independent step of cytokinesis. *Nature* **554**, 528-532, doi:10.1038/nature25506 (2018).

6 Veiga, H., Jorge, A. M. & Pinho, M. G. Absence of nucleoid occlusion effector Noc impairs formation of orthogonal FtsZ rings during *Staphylococcus aureus* cell division. *Mol. Microbiol.* **80**, 1366-1380, doi:10.1111/j.1365-2958.2011.07651.x (2011).

7 Jorge, A. M., Hoiczyk, E., Gomes, J. P. & Pinho, M. G. EzrA contributes to the regulation of cell size in Staphylococcus aureus. *PLoS One* **6**, e27542, doi:10.1371/journal.pone.0027542 (2011).

8 Turner, R. D. *et al.* Peptidoglycan architecture can specify division planes in *Staphylococcus aureus*. *Nat. Commun.* **1**, 26, doi:10.1038/ncomms1025 (2010).

9 Monteiro, J. M. *et al.* Cell shape dynamics during the staphylococcal cell cycle. *Nat Commun* **6**, 8055, doi:10.1038/ncomms9055 (2015).
