# Supplementary material for: Reassessment of the distinctive geometry of Staphylococcus aureus cell division
Source: Nat Commun. 2020 Aug 14;11:4097. doi: 10.1038/s41467-020-17940-9 (PMC7427965; doi:10.1038/s41467-020-17940-9)
Supplement: Supplementary file 2 — Description of Additional Supplementary Files [file 41467_2020_17940_MOESM2_ESM.pdf]

Description of Additional Supplementary Files

File Name: Supplementary Movie 1

Description: 3D rendering of EzrA-GFP Z-stack images, obtained from COL EzrAsGFP cells, rotated to allow visualization of the angle between the divisomes of attached sister cells.

File Name: Supplementary Software

Description: The included source code was used to measure the angle between the division plane of sister cells. The code was written in Python3 and takes as input kymographs generated by drawing a line parallel to the flat region of the divisome for each sister cell. The software then isolates the fluorescence signal in each kymograph and calculates the angle relative to the image acquisition plane by extracting the first principal component of the isolated pixels coordinates. After calculating the angle for each kymograph, it then calculates the absolute difference between the two angles, corresponding to the angle of divisomes in two sister cells.
