# Supplementary material for: Reassessment of the distinctive geometry of Staphylococcus aureus cell division
Source: Nat Commun. 2020 Aug 14;11:4097. doi: 10.1038/s41467-020-17940-9 (PMC7427965; doi:10.1038/s41467-020-17940-9)
Supplement: Supplementary file 4 — Supplementary Software [file 41467_2020_17940_MOESM4_ESM.zip › Source Code/example/report.html]

title


Cell Name | Line1 | Kym1 | Kym1 Filtered | Kym1 Angle | Line2 | Kym2 | Kym2 Filtered | Kym2 Angle | Angle Difference || Cell\_10 |  |  |  | -44.61 |  |  |  | -79.81 | 35.20 |
| Cell\_12 |  |  |  | 41.29 |  |  |  | -41.98 | 83.27 |
