# Supplementary figures and images for: Reassessment of the distinctive geometry of Staphylococcus aureus cell division
Source: Nat Commun. 2020 Aug 14;11:4097. doi: 10.1038/s41467-020-17940-9 (PMC7427965; doi:10.1038/s41467-020-17940-9)

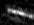

Supplement: Supplementary file 4 — Supplementary Software [file 41467_2020_17940_MOESM4_ESM.zip › Source Code/example/Cell_10/kym1.tif]

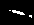

Supplement: Supplementary file 4 — Supplementary Software [file 41467_2020_17940_MOESM4_ESM.zip › Source Code/example/Cell_10/kym1_filtered.png]

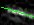

Supplement: Supplementary file 4 — Supplementary Software [file 41467_2020_17940_MOESM4_ESM.zip › Source Code/example/Cell_10/kym1_w_line.png]

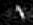

Supplement: Supplementary file 4 — Supplementary Software [file 41467_2020_17940_MOESM4_ESM.zip › Source Code/example/Cell_10/kym2.tif]

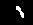

Supplement: Supplementary file 4 — Supplementary Software [file 41467_2020_17940_MOESM4_ESM.zip › Source Code/example/Cell_10/kym2_filtered.png]

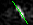

Supplement: Supplementary file 4 — Supplementary Software [file 41467_2020_17940_MOESM4_ESM.zip › Source Code/example/Cell_10/kym2_w_line.png]

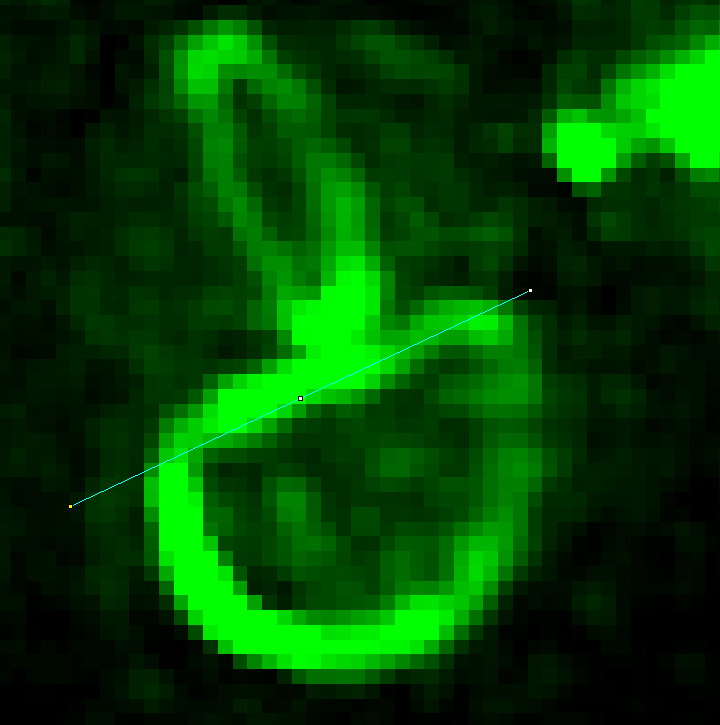

Supplement: Supplementary file 4 — Supplementary Software [file 41467_2020_17940_MOESM4_ESM.zip › Source Code/example/Cell_10/kymline1.png]

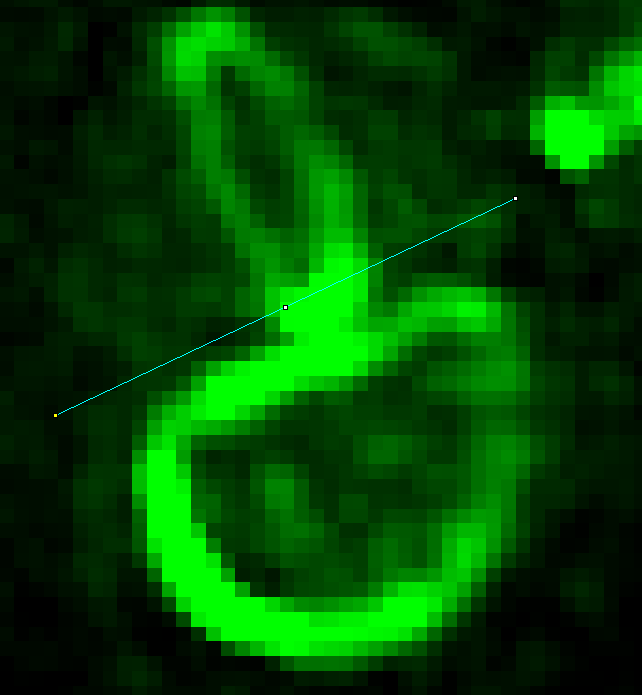

Supplement: Supplementary file 4 — Supplementary Software [file 41467_2020_17940_MOESM4_ESM.zip › Source Code/example/Cell_10/kymline2.png]

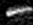

Supplement: Supplementary file 4 — Supplementary Software [file 41467_2020_17940_MOESM4_ESM.zip › Source Code/example/Cell_12/kym1.tif]

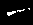

Supplement: Supplementary file 4 — Supplementary Software [file 41467_2020_17940_MOESM4_ESM.zip › Source Code/example/Cell_12/kym1_filtered.png]

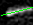

Supplement: Supplementary file 4 — Supplementary Software [file 41467_2020_17940_MOESM4_ESM.zip › Source Code/example/Cell_12/kym1_w_line.png]

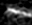

Supplement: Supplementary file 4 — Supplementary Software [file 41467_2020_17940_MOESM4_ESM.zip › Source Code/example/Cell_12/kym2.tif]

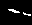

Supplement: Supplementary file 4 — Supplementary Software [file 41467_2020_17940_MOESM4_ESM.zip › Source Code/example/Cell_12/kym2_filtered.png]

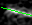

Supplement: Supplementary file 4 — Supplementary Software [file 41467_2020_17940_MOESM4_ESM.zip › Source Code/example/Cell_12/kym2_w_line.png]

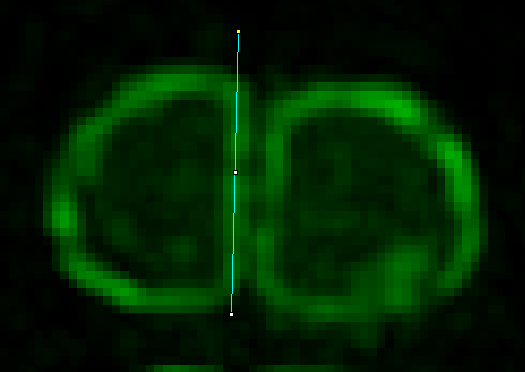

Supplement: Supplementary file 4 — Supplementary Software [file 41467_2020_17940_MOESM4_ESM.zip › Source Code/example/Cell_12/kymline1.png]

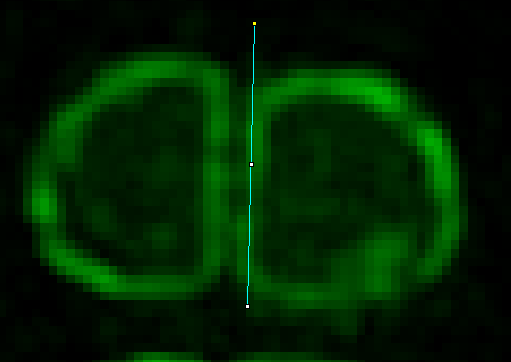

Supplement: Supplementary file 4 — Supplementary Software [file 41467_2020_17940_MOESM4_ESM.zip › Source Code/example/Cell_12/kymline2.png]
